# Design and testing of a centrifugal fluidic device for populating microarrays of spheroid cancer cell cultures

**DOI:** 10.1186/s13036-020-0228-6

**Published:** 2020-03-12

**Authors:** Warren Weisler, Samuel Miller, Shaphan Jernigan, Gregory Buckner, Matthew Bryant

**Affiliations:** Department of Mechanical and Aerospace Engineering, North Carolina State University, Raleigh, NC 27695-7910 USA

## Abstract

**Background:**

In current cancer spheroid culturing methods, the transfer and histological processing of specimens grown in 96-well plates is a time consuming process. A centrifugal fluidic device was developed and tested for rapid extraction of spheroids from a 96-well plate and subsequent deposition into a molded agar receiver block. The deposited spheroids must be compact enough to fit into a standard histology cassette while also maintaining a highly planar arrangement. This size and planarity enable histological processing and sectioning of spheroids in a single section. The device attaches directly to a 96-well plate and uses a standard centrifuge to facilitate spheroid transfer. The agar block is then separated from the device and processed.

**Results:**

Testing of the device was conducted using six full 96-well plates of fixed Pa14C pancreatic cancer spheroids. On average, 80% of spheroids were successfully transferred into the agar receiver block. Additionally, the planarity of the deposited spheroids was evaluated using confocal laser scanning microscopy. This revealed that, on average, the optimal section plane bisected individual spheroids within 27% of their mean radius. This shows that spheroids are largely deposited in a planar fashion. For rare cases where spheroids had a normalized distance to the plane greater than 1, the section plane either misses or captures a small cross section of the spheroid volume.

**Conclusions:**

These results indicate that the proposed device is capable of a high capture success rate and high sample planarity, thus demonstrating the capabilities of the device to facilitate rapid histological evaluation of spheroids grown in standard 96-well plates. Planarity figures are likely to be improved by adjusting agar block handling prior to imaging to minimize deformation and better preserve the planarity of deposited spheroids. Additionally, investigation into media additives to reduce spheroid adhesion to 96-well plates would greatly increase the capture success rate of this device.

## Background

**Fig. 1:**
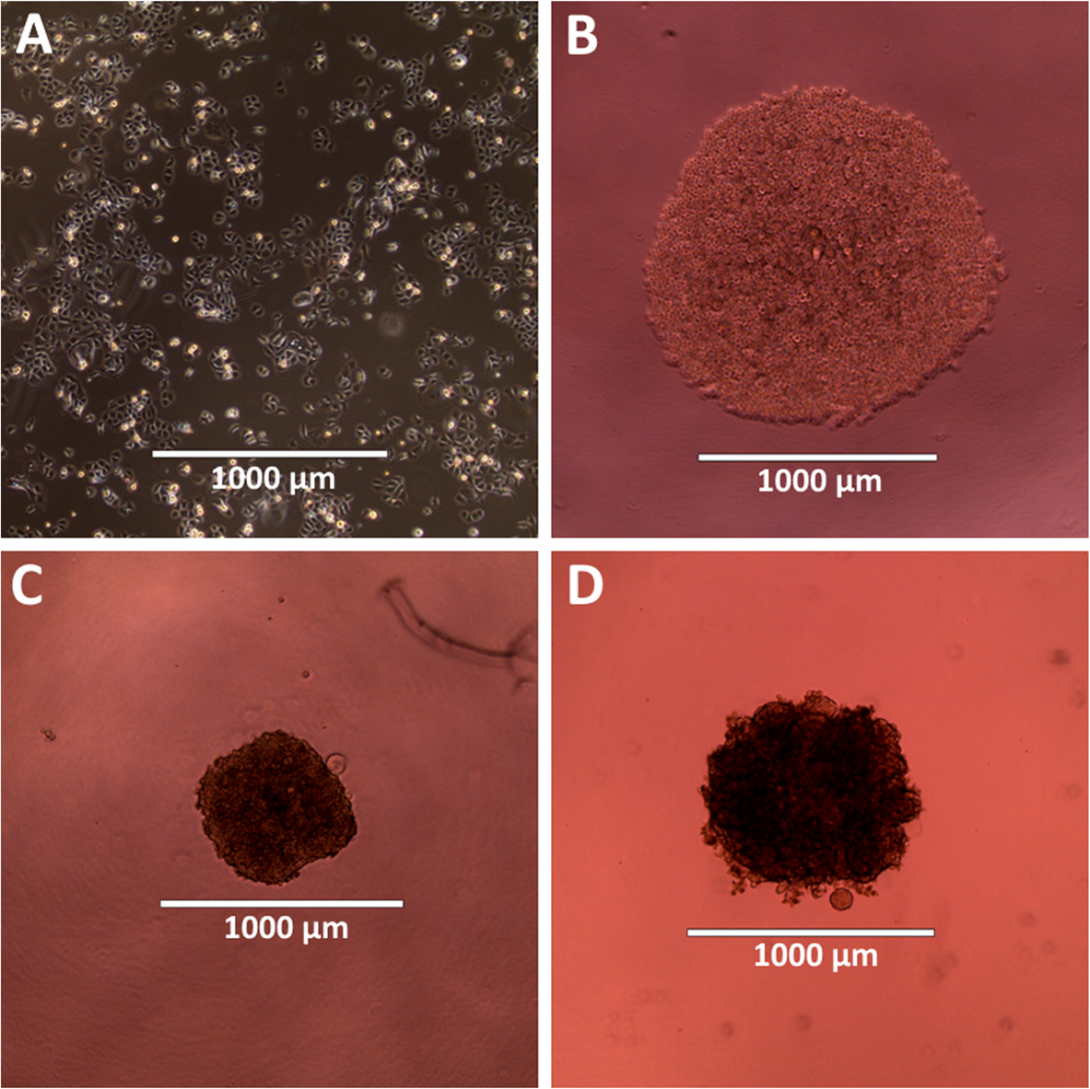
Pa14C pancreatic cancer cells. **a** Cells in 2D culture. **b** Cells seeded into a round bottom plate and centrifuged to aggregate. **c** Cells formed into a spheroid 2 days after seeding. **d** Spheroid 14 days after seeding. Scale bar represents 1000 μm in all images

Cell culturing is a key experimental tool in the study of solid tumor biology, pharmacology, and the search for more effective cancer treatments. Two-dimensional (2D) in vitro cell culturing, in which cells are grown on flat glass or plastic substrates (Fig. 1a), gained widespread acceptance after its introduction in the early twentieth century, and remains the most common choice for drug screening studies partly due to its well-developed compatibility with high-throughput and automated methods. Unfortunately, several factors limit the accuracy with which 2D cultures model in vivo tissues, leading to the development of phenotypes in 2D cultures that vary significantly from cells in vivo [1, 2]. These factors include: differences in strain distributions for cells grown on 2D rigid substrates versus 3D environments [3–6]; differences in mass transport, which limits cellular access to oxygen, nutrients, and soluble factors; and the lack of molecular gradients [7–9]. For these reasons, drug screens based on 2D cell cultures can lead to misleading or non-predictive results [10].

Three-dimensional (3D) in vitro cell cultures (Fig. 1c and d) are finding increased application in pathobiological and pharmacological studies of solid tissues. “Simple spheroid” cultures derived from single established cell lines can be readily grown using round bottom 96-well plates molded from ultra-low attachment substrates. Because these 3D cultures more closely resemble in vivo tissues than their 2D counterparts, they may provide more accurate modeling of in vivo tissues. For example, spheroid cultures have been demonstrated to more accurately predict the magnitude of in vivo tumor response in KRAS driven cancers [11].

Microscopic analysis of 3D cell cultures is of particular interest, as it enables spatial characterization and mapping of biomarkers associated with biological function (e.g. metabolic activities) and responses to targeted therapies. A limiting factor is the histologic analysis of 3D cultures using existing tools and techniques: the manual process is time-consuming, inefficient, and cannot compete with the throughput of robotic systems used in the screening of 2D microwell plate formats.

We have developed a prototype device, the centrifugal funnel array, that enables simultaneous transfer of 96 spheroid cultures from standard 96-well plates to agar-encapsulated microarrays for histological processing and analysis. This technology has the potential to expedite the preparation and analysis of 3D spheroid cultures enabling high-throughput drug screens, better predictions of solid tissue responses, and more rapid development of therapeutics. Because the specimens maintain their relative position and registry within the array during transfer, the effects of experimental conditions in each well can be precisely tracked. Each microarray can then be embedded in paraffin and sectioned as a single specimen, enabling simultaneous microscopic analysis of all 96 spheroids.

**Fig. 2:**
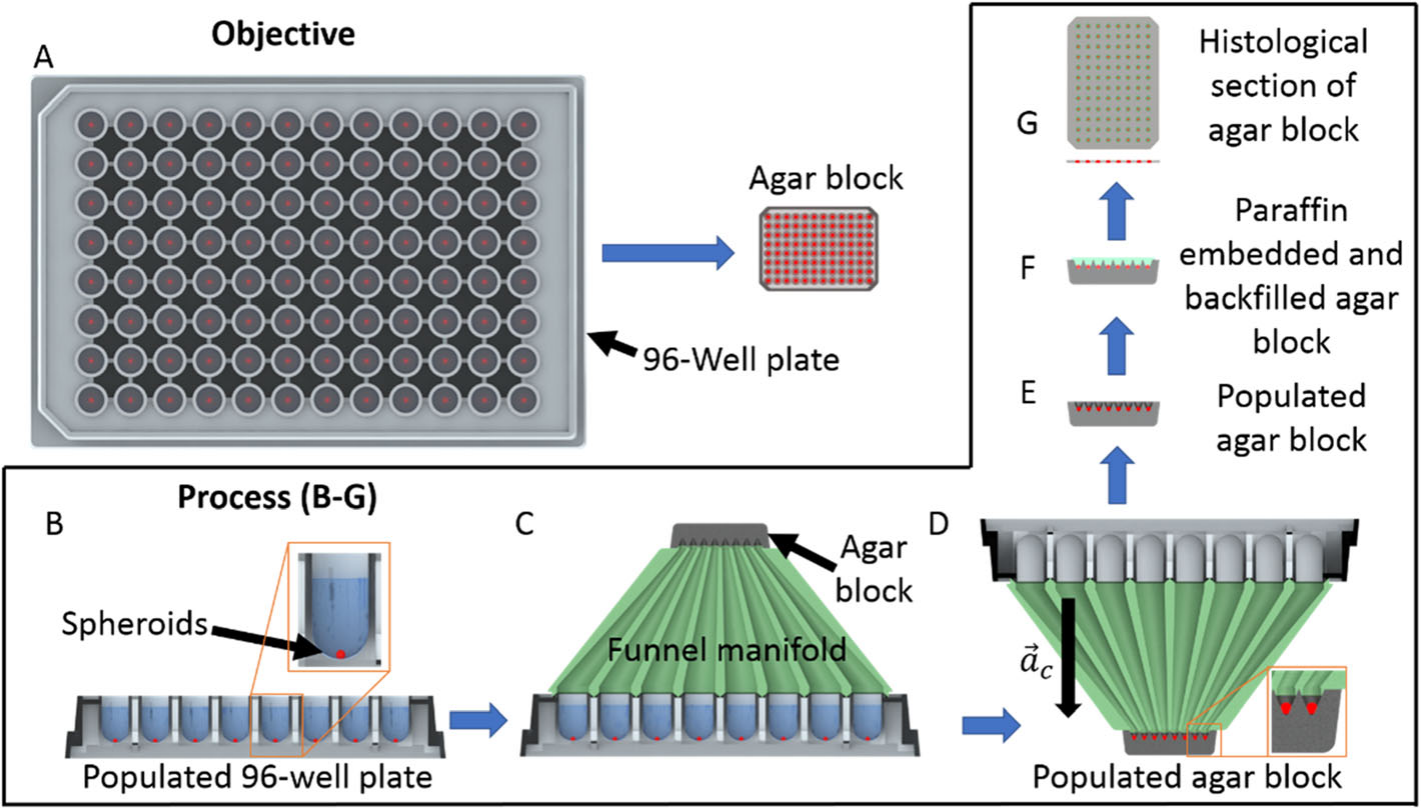
Conceptual design and operation. **a** Objective of proposed device showing the relative sizing of the 96-well plate and agar block. **b** Spheroids (red) grown in a 96-well plate (light grey) with liquid medium (blue). **c** Funnel manifold (green) with attached agar block (dark grey) mounted on a 96-well plate. **d** Centrifuge process using centripetal acceleration to move spheroids into the agar block. **e** Populated agar block removed from funnel manifold. **f** Agar block encapsulated and paraffin embedded after backfilling with liquid agar (light green). **g** Top and side view of a histological section of the agar block. Note: Not to scale, spheroids enlarged for clarity

A simple concept of operations for the proposed device is shown in Fig. 2. The functional objective is to automate the transfer of spheroids from a 96-well plate to an agar block suitable for histological processing. The position of each spheroid within the 96-well array should be preserved, while reducing the array dimensions as shown in Fig. 2a. The agar block can then be histologically processed and sectioned, enabling simultaneous microscopic imaging of all 96 spheroids on a single slide. The proposed process begins with a 96-well plate containing formaldehyde-fixed spheroids (Fig. 2b). A micro-Funnel Manifold (μFM) consisting of 96 tapered converging funnels is attached to the 96-well plate along with the molded agar block containing 96 microwells (Fig. 2c). The device is then inverted and placed in a standard clinical benchtop centrifuge, which facilitates spheroid transfer from the 96-well plate to the agar block (Fig. 2d). The populated agar block is then removed from the device (Fig. 2e). Next, the transferred spheroids are fully encapsulated by backfilling the agar block with liquid agar. The backfilled block then undergoes standard paraffin embedding and histological processing (Fig. 2f). Microtome sections of the block are mounted on glass slides such that all 96 spheroids can be examined on a single slide (Fig. 2g).

This paper presents the testing and evaluation of the centrifugal funnel array. The device was tested using full 96-well plates of fixed Pa14C spheroids. The transfer success rate for each plate and well was recorded following centrifugation. The resulting agar blocks were imaged using confocal laser scanning microscopy (CLSM) to determine the 3D location of each spheroid within the block. Finally, the spheroid location and size data from CLSM were used to quantitatively assess the planarity of the deposited specimens.

## Results

**Fig. 3:**
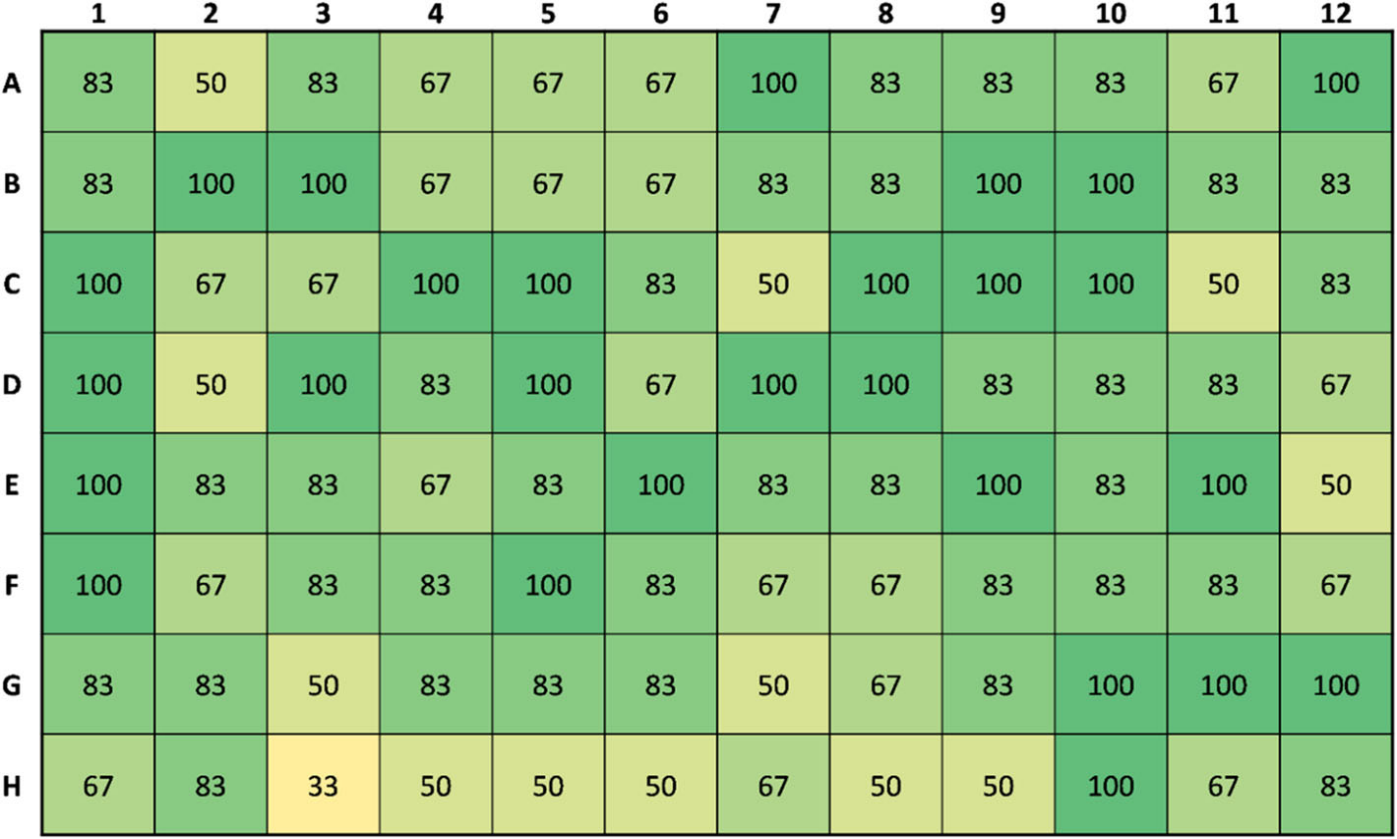
Spheroid capture success rate. The success rate out of 100% is shown for each well of a Costar 7007 ultra-low attachment round bottom 96-well plate (*N* = 6). The average success rate over all 6 plates was 80 ± 11%

The centrifugal funnel array was tested with a total of six full 96-well plates of fixed Pa14C spheroids. Four of these six plates were also optically sectioned using the CLSM. For these six plates, the average spheroid transport success rate was 80 ± 11%. In other words, on average 80% of all spheroids were successfully transferred from the 96-well plate and captured in the agar receiver block for each test. The transport success rate was also recorded for each well on the 96-well plate. These results are summarized in Fig. 3 which indicates the average success rate for each well (*N* = 6).

**Fig. 4:**
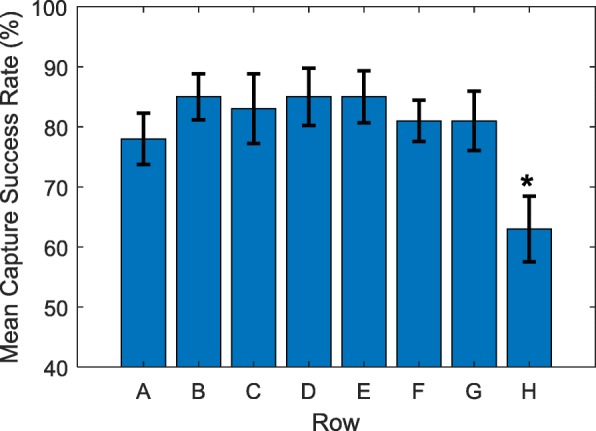
Mean capture success rate for each row. Error bars indicate standard error. (*) the mean success rate of this row is significantly different (***p <*0.01**) from the mean success rate for all rows

**Fig. 5:**
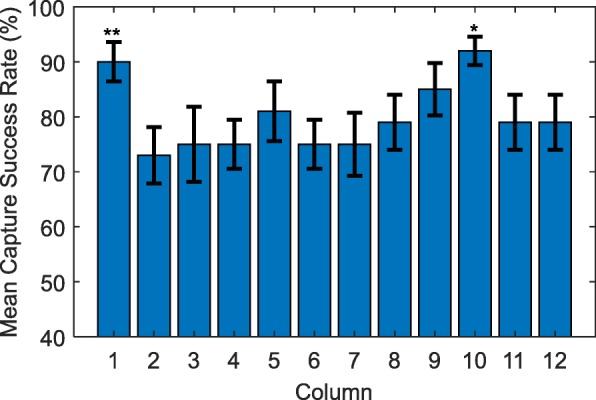
Mean capture success rate for each column. Error bars indicate standard error. (*) the mean success rate of this column is significantly different (***p <*0.01**) from the mean success rate for all columns. (**) ***p ≈*0.01** for this column

Figures 4 and 5 show spheroid capture success rates averaged across rows and columns, respectively. In these figures, error bars indicate the standard error of the mean. Bars marked with an asterisk indicate the rows/columns that have mean capture success rates which are significantly different from the total mean capture success rate over all wells.

**Fig. 6:**
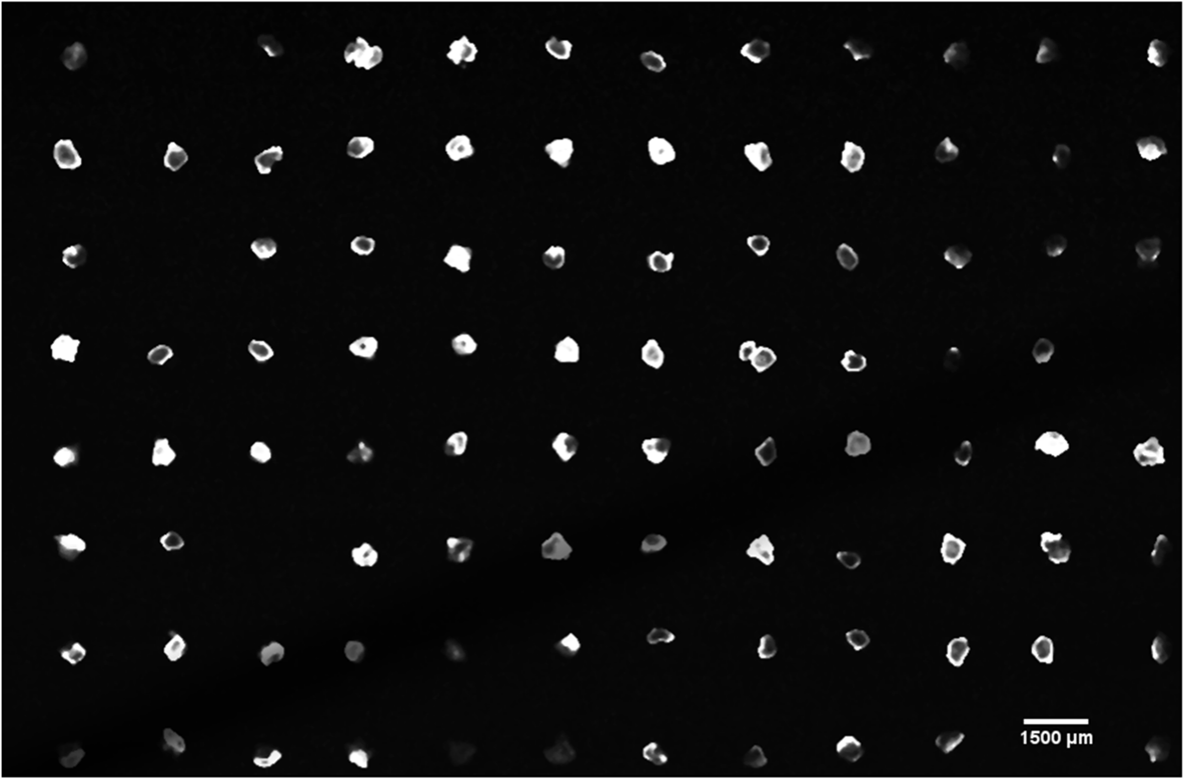
Optical section from CLSM. This 30 μm thick section shows a cross section of each spheroid captured (91 out of 96 spheroids were captured in this sample). Variations in the light intensity of the spheroids are due to their depth relative to the section plane. Higher intensity spheroids are deeper relative to the section plane than lower intensity spheroids

The spheroid distribution planarity was evaluated for four of the six plates by using CLSM as stated before. Figure 6 shows an optical section from a receiver block containing 91 out of 96 spheroids. When imaging a plane passing through the far side of a spheroid (with respect to the objective), the fluorescent signal must pass through a large portion of spheroid tissue. This results in signal degradation, causing a reduction in image intensity as seen in Fig. 6. This figure shows that a 30 μm thick section from this sample contains a cross section from all 91 spheroids in the agar receiver block.

The fit plane and normalized distance statistics for each sample are shown in Table 1. The centroid fit plane is defined by the coefficients *p*_0_, *p*_*x*_, and *p*_*y*_. Here, *p*_0_ represents z-height of the fit plane from the bottom surface of the agar block, *p*_*x*_ indicates the slope of the plane in the x-direction, and *p*_*y*_ indicates the slope of the plane in the y-direction. The slope of the plane can be interpreted as a measure of how parallel the spheroid plane is to the bottom surface of the agar block. The mean normalized distance to the plane averaged over all four samples was 0.27 ± 0.064.

**Table 1:** Spheroid distribution planarity statistics

	Centroid Plane Fit			Normalized Distance		
	*z* = *p*_0_ + *p*_*x*_*x* + *p*_*y*_*y* (*μm*)			${d}_i/{\overline{r}}_i$		
Sample	*p*_0_	*p*_*x*_	*p*_*y*_	Mean	Median	Max
1	812.76	−0.006266	− 0.01693	0.2071	0.1805	0.6915
2	888.18	−0.00760	−0.01678	0.3508	0.2762	1.1007
3	799.91	−0.011548	−0.00528	0.2306	0.2053	0.6558
4	512.34	−9.09E-05	−0.01062	0.2813	0.2218	1.2853

**Fig. 7:**
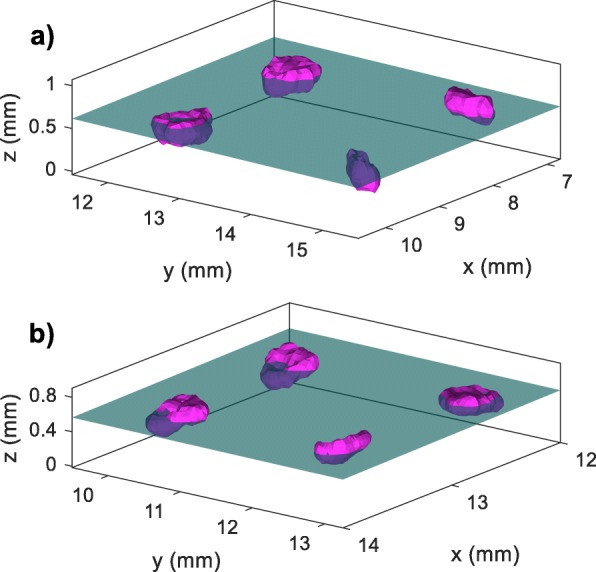
Plane passing through a selection of spheroids from Sample 2 (**a**) and Sample 3 (**b**). This figure shows a selection of 4 spheroids and the corresponding fit plane calculated for the entire array. The spheroid surfaces (magenta) are generated from the CLSM image stacks

Fig. 7 illustrates how well the fit plane sections the receiving blocks. In this figure, (a) shows a selection of four spheroids from Sample 2 while (b) shows a selection from Sample 3. The selection in (a) was chosen to show the most out-of-plane spheroid through which the plane only sections a small piece. This is reflected in the maximum normalized distance of 1.1 for this sample.

**Fig. 8:**
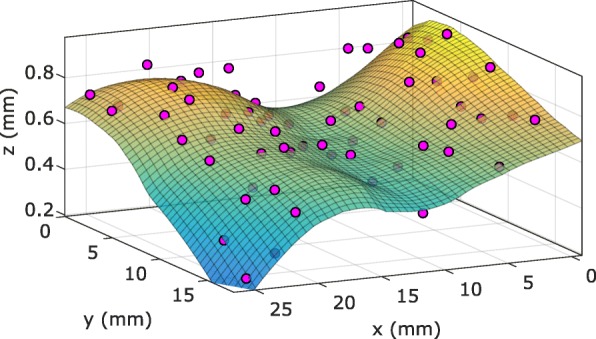
Spheroid centroid distribution for sample 2 using the same coordinate system as that used in Fig. 7a. The magenta markers represent centroid locations and do not indicate spheroid volume. While not indicative of sectionability, a locally weighted regression was added to highlight the non-planar trends in the distribution (***R***^**2**^***=*0.87**)

To show spheroid distribution trends for an entire sample, spheroid centroids were fit with a locally weighted regression. Fig. 8 shows the spheroid centroids (magenta circles) and their corresponding regression (*R*^2^ = 0.87) for Sample 2. Here, the z-axis scale was zoomed in to better show variations in spheroid position. Note that the circular markers in Fig. 8 do not represent spheroid shape or size.

## Discussion

Some notable trends can be inferred from Figs. 3, 4 and 5, the most obvious being the relatively low success rate for wells H3 through H9, which includes the well with the lowest average success rate: H3. Figure 4 shows that on average, the success rate of row H is significantly lower (*p* < 0.01) than the mean success rate of 80%. This effect is possibly explained by the technique used to invert the device prior to centrifugation. For example, if the device is rotated around a particular column or row then the effects of the motion on the samples in those wells may be different. In contrast, Fig. 5 indicates that no columns had a significantly lower success rate than the mean, however Column 10 was significantly higher than the mean (*p* < 0.01).

After each test, the device was inspected for uncaptured spheroids. An overwhelming majority of the spheroids which were not captured in the receiver block remained inside the 96-well plate. This also shows that the device itself is providing a reliable passage from the 96-well plate to the receiver block. As stated before, this effect may be the result of the technique used to invert the device. While fine tuning this technique (e.g. by increasing or decreasing agitation) could be done to improve the transfer between the well plate and the device, robustness to variations in end-user technique is a priority.

One proposed method of addressing this issue is by using media additives. It is unclear as to the exact reason some spheroids adhere to the plate, however some possible explanations include surface tension effects, surface adsorption of biomolecules, friction, and electrostatic forces. There is a considerable amount of research in the area of surface modifications for reducing cell and tissue adhesions to engineered materials [12, 13]. This information can help in the design of surface coatings for the μFM but has limited applications to the use of standard off-the-shelf 96-well plates which would not have this coating. This makes media additives an attractive option for modifying spheroid surface interactions. Possible additives for future research include biological lubricants such as those found the synovial fluid [14], water-based lubricants such as methylcellulose, ionic and anionic detergents, and various poloxamers [15].

For spheroids that were successfully transferred, the results in Table 1 show that the optimal section plane passes through the spheroids within approximately 27% of their mean radius. The standard deviation in the normalized distance to plane over all four samples was 0.064 which suggests repeatable planarity results between samples. Thus, it appears that the spheroids are mostly distributed in a planar fashion. However, the maximum normalized distance for Samples 2 and 4 are greater than 1. This indicates that there is at least one spheroid that may not be sectioned by the fit plane. This also points to a limitation of these statistics: the “sphericity” of the spheroids affects the strength of the conclusion. It is not entirely true to say that a normalized distance of greater than 1 means that a spheroid was not sectioned by the fit plane. As demonstrated by Fig. 7a, the spheroid sitting well below the plane is more of an ellipsoid. This indicates that the mean radius underestimates the size of the spheroid along its major axis. Along its minor axes, the mean radius overestimates the size. Figure 7b shows spheroids from a sample with a maximum normalized distance of well under 1. This selection shows more uniformity in the size of the cross sections generated by the fit plane.

Upon further investigation of the CLSM results, it became apparent that some of the samples had spheroid distributions that were more parabolic than planar. The regression in Fig. 8 shows a depression running through the sample parallel to the y-axis. In fact, the selection from Fig. 7a shows how spheroids in this depression relate to the fit plane. In Fig. 8, *x* = 10 mm corresponds with the bottom of the depression in this range of y-positions. This anomaly in the distribution could be caused by bending of the sample during preparation or handling that remained through imaging. In addition, during imaging the agar receiver block was placed on a glass coverslip. Thus, if the surface contacting the coverslip was not perfectly flat the block would deform from its original shape. The planarity statistics are likely to be improved by adjusting the sample preparation and handling procedures, such as using a vibratome to plane the surface of the agar block prior to mounting on the glass coverslip.

These testing results helped identify a specific area of the methodology which needs improvement: transfer of spheroids out of the 96-well plate. In some cases, spheroids adhered to the inside of their wells accounted for over 85% of the failed captures. This prompts further investigation into the use of media additives as previously discussed or using plates made of a different material. The planarity results indicate that the spheroids are largely being deposited in a plane, however some outliers still exist. Further analysis of the CLSM results shows that some samples have spheroid distributions with parabolic trends. As mentioned earlier, this is likely the result of an uneven agar surface or bending of the receiving block. Embedding alignment features in a plane in the agar could allow for correction of sample deformation during CLSM, however more refined sample preparation and handling should abate this problem without the need for more complex image analysis techniques.

## Conclusions

This paper has presented the development and testing of a novel device for the transfer of spheroid cultures from a 96-well plate to a histology cassette, while maintaining the same relative location of the spheroids before and after the transfer. The device uses a centrifuge to move the spheroids from the 96-well plate through funnels and into an agar receiver block, forming an agar gel embedded microarray which can then be processed and imaged. Device testing revealed that on average 80% of fixed Pa14C spheroids were successfully deposited in the agar receiver block. The planarity of the spheroids in the agar block was evaluated using a confocal laser scanning microscope which demonstrated that, on average, spheroids were sectioned within 27% of their mean radius. This shows that the vast majority of deposited spheroids could be examined in a single section. While the device was successful at capturing most spheroids, the success rate could be improved by focusing on the issue of spheroid adhesion to the 96-well plate. This prompts future studies into media additives to aid in spheroid handling.

This device could reduce the time required to evaluate spheroids from 96-well plates by not only reducing the labor intensive task of extracting spheroids individually from plates, but also by allowing the spheroids to be processed in a single histology cassette and imaged as a single specimen. Additionally, this parallel processing has the potential to reduce slide-to-slide and spheroid-to-spheroid variation introduced by manual serial processing and imaging. Future work in this area should be conducted to characterize the benefits and effects of various 3D culture handling methods on the outcome of downstream processes.

## Methods

### Materials

Corning Costar 7007 ultra-low attachment round bottom 96-well plates and phosphate buffered saline (PBS) were obtained from Corning (Corning, NY). Dulbecco’s Modified Eagle Medium (DMEM), penicillin-streptomycin, and HCS CellMask Deep Red stain were acquired from Thermo Fisher Scientific (Waltham, MA). Laminin-I was purchased from Trevigen (Gaithersburg, MD). For fixation, 8% paraformaldehyde (PFA) solution was obtained from Electron Microscopy Sciences (Hatfield, PA). Parafilm “M” was obtained from Bemis (Oshkosh, WI). Fetal bovine serum (FBS), Agar powder, and Triton X-100 were purchased from Sigma-Aldrich (St. Louis, MO). The High-Density Polyethylene (HDPE) inserts were acquired from Hero Glue (Las Vegas, NV). The neoprene gasket, absorbent mat, and nylon mesh for the containment base were obtained from McMaster-Carr (Elmhurst, IL). All other device components were fabricated with a Stratasys (Rehovot, Israel) Objet 30 Polyjet 3D printer using VeroWhitePlus acrylic photopolymer and support material. Centrifugation was conducted using a Sorvall Legend RT obtained from Thermo Fisher Scientific (Waltham, MA). For confocal microscopy, a Zeiss (Oberkochen, Germany) LSM 880 was used.

### Cell culture

The device was tested with fixed spheroids cultured from the Pa14C pancreatic adenocarcinoma cell line [16]. Pa14C cells were provided by A. Maitra (MD Anderson Cancer Center). Cells were grown in a growth medium consisting of DMEM supplemented with 10% FBS and 1% penicillin-streptomycin. Cells were maintained at 37 °C, 5% CO_2_. Cells were maintained at low passage (less than 12).

### Spheroid generation and fixation

Cells were harvested and seeded into Corning Costar 7007 ultra-low attachment round bottom 96-well plates at a density of 3000 cells per well in 50 μL of liquid medium. This medium consisted of growth medium supplemented with 5% laminin-I. Laminin-I was used because it has been shown to promote E-cadherin expression which promotes cell-cell contacts, rather than contacts with the substrate. The addition of laminin-I to 3D culture systems has also been shown to promote more cuboidal clustered cell morphology [17]. Plates were then centrifuged at 300×g for 3 min and allowed to incubate at 37 °C. After 3 days, an additional 150 μL of growth medium was added to each well. Media was replenished 7 days post seeding. Spheroids were cultured for a total of 10 to 14 days prior to fixation.

Spheroids were fixed by removing half of the culture medium from each well and replacing with an equal amount of 8% PFA solution. The spheroids in PFA were then refrigerated at 4 °C overnight. The following day, spheroids were washed in PBS by transferring each spheroid from the PFA solution to a 96-well plate containing 150 μL of PBS. Next, the PBS was decanted from each well leaving each spheroid behind. Finally, 150 μL of fresh PBS was added to each well. The fixed spheroid plates were wrapped in parafilm and stored at 4 °C for future use.

### Agarose receiver block preparation

**Fig. 9:**
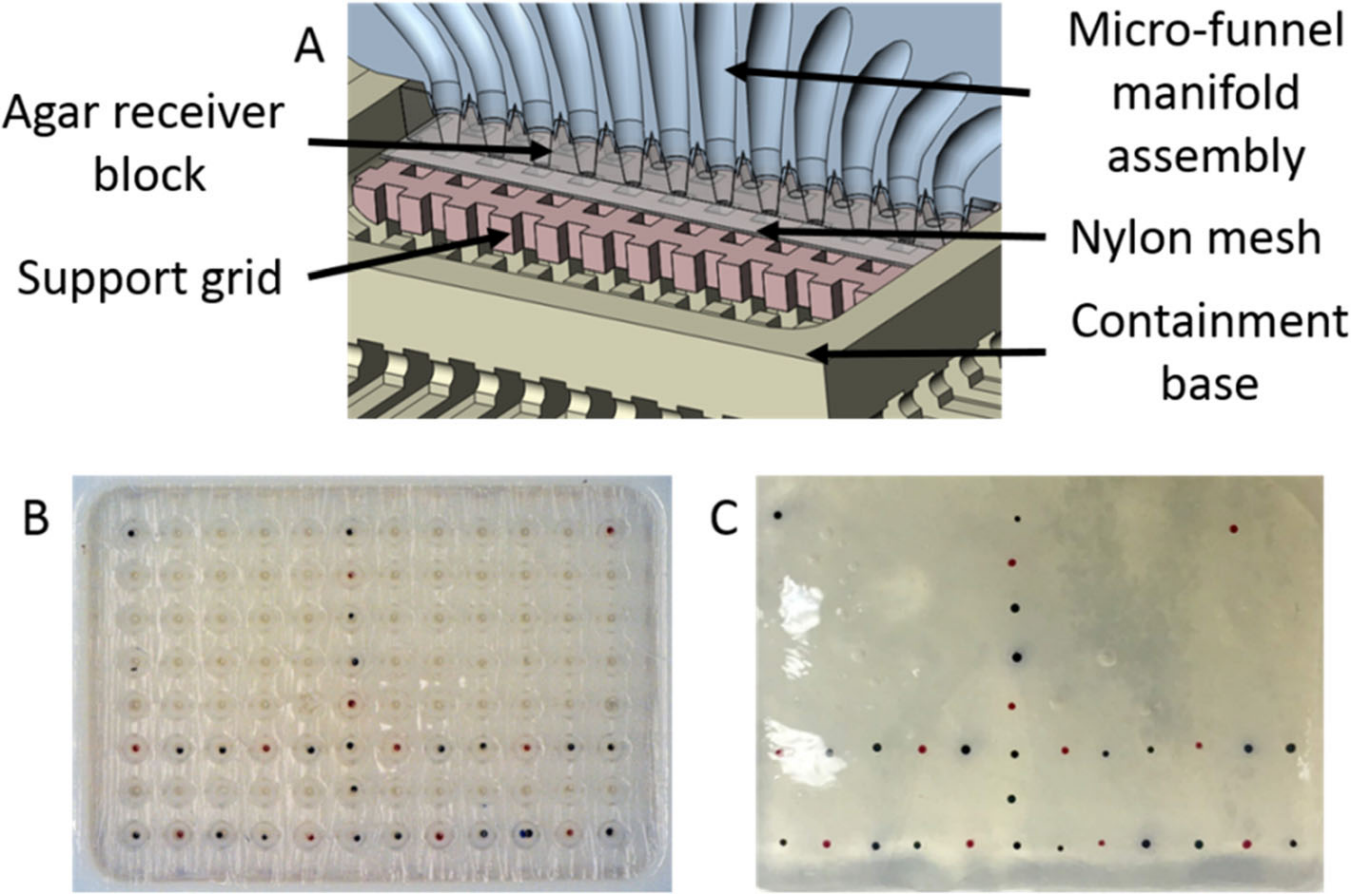
Agar receiver block used with micro-funnel manifold assembly. **a** CAD rendering showing the bottom of the micro-funnel manifold assembly (blue), agar receiver block (translucent), nylon mesh (grey), support grid (pink), containment base (tan). **b** Dyed microspheres deposited in the agar receiver block after centrifugation. Note only two rows, one column, and four corners were populated in the 96-well plate. **c** Dyed microspheres encapsulated in agar receiver block

Work by Ivanov et al. [18] demonstrated that an array of 11 by 6 spheroids deposited in an agar gel block could be made to fit in a histology cassette by manual micropipetting. A similar concept was used here to ensure that a full plate of 96 (12 × 8) spheroids could be successfully deposited by the μFM and encapsulated in an agar block of these dimensions. While the agar blocks in [18] used blind holes for spheroid capture, the agar blocks used here were cast with through holes and placed on top of a 100 μm nylon mesh (Fig. 9a). This allowed captured spheroids to be separated from solution prior to agar encapsulation (Fig. 9b and c).

Agar receiver blocks were prepared by first mixing a 4% w/v solution of agar powder and phosphate buffered saline in a conical centrifuge tube. The tube was then placed in a temperature controlled water bath at 85 °C until fully dissolved. Agar solutions were then kept in liquid form at 40 °C prior to casting. Blocks were cast by pouring approximately 5 mL of liquid agar solution into a custom 3D printed mold and allowed to rest for 10 to 15 min. After fully gelled, the blocks were extracted from the mold and stored in PBS at 4 °C until used.

### Device operation

The physical dimensions and mass of the prototype device were limited by the testing centrifuge bucket volume and mass capacity, respectively. A device geometry was established which met this geometric constraint while adequately mating with the 96-well plate. Indexing features were used on the μFM top to ensure that it aligned precisely with the 96-well plate. Next, the interior length and width dimensions of a standard histology cassette, 3.0 cm and 2.5 cm, respectively, were used to establish the diameters and spacing of the funnel outlets in the μFM bottom. Based on an average spheroid diameter of 500 μm, these dimensions resulted in an outlet diameter of 2200 μm with a center-to-center spacing of 3800 μm.

**Fig. 10:**
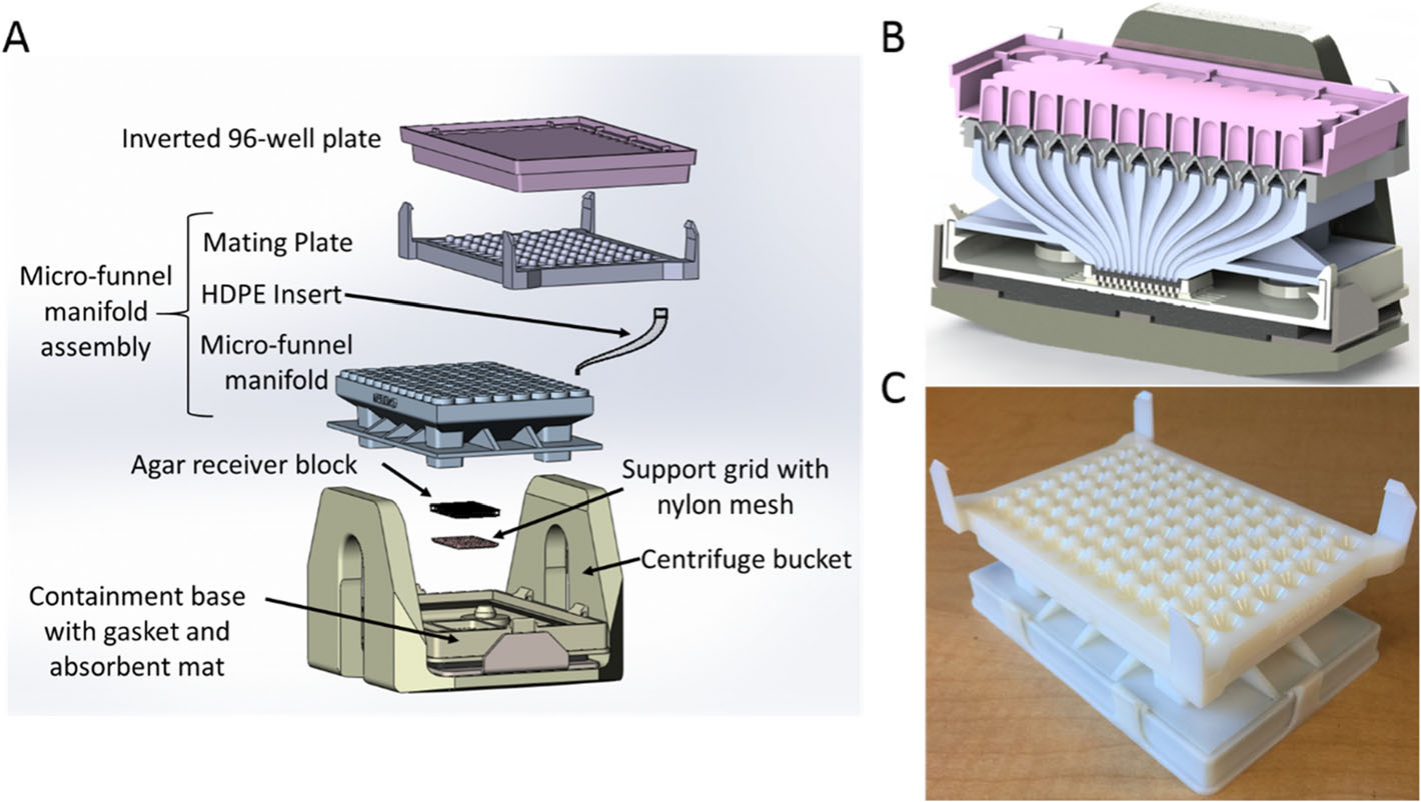
Overview of μFMA construction. **a** Exploded view of device in a centrifuge bucket (gasket and absorbent mat not shown). **b** Cross section view of fully assembled device in centrifuge bucket (gasket and absorbent mat not shown). **c** Photo of assembled device ready to mate with a 96-well plate

The components of the fabricated prototype are listed from the top downward (Fig. 10a): 96-well plate, mating plate, HDPE inserts, μFM, agar receiver block, nylon mesh, support grid, containment base, an absorbent mat, and a gasket. The mating plate, μFM, support grid, and containment base were all 3D printed. Tapered HDPE inserts used between the mating plate and the μFM provided a smooth continuous passage to mitigate friction acting on spheroids during device operation. The μFM equipped with HDPE inserts and epoxied to the mating plate forms the micro-Funnel Manifold Assembly (μFMA), which is used in operation as a single component. Figure 10b shows a cross section view of the assembled device. Here, the geometry and feature size of each funnel necessitates the use of high resolution additive manufacturing techniques.

Device operation begins by positioning the agar block, nylon mesh, support grid, absorbent pad, and gasket in the containment base. The manifold assembly is then mated with the containment base. Next, the entire assembly (Fig. 10c) is inverted and clipped onto the upright 96-well plate containing fixed Pa14C spheroids. The device, now mated to the 96-well plate, is then slowly inverted and placed in the centrifuge bucket, utilizing a counterweight as needed. After centrifuging at 300×g for 5 min, the device is removed and the manifold assembly is detached from the containment base. Liquid agar (4% w/v previously prepared) at 40 °C is poured over the agar receiver block, filling the through holes and encapsulating the transferred spheroids. After cooling for 10 to 15 min, the agar receiver block was removed from the containment base and placed in a petri dish containing PBS. From here, the nylon mesh was removed from the block and replaced with a thin layer of liquid agar. Once the final layer of agar gelled, the finished receiver block with encapsulated spheroids was stored in PBS at 4 °C in preparation for downstream processing.

### Optical sectioning and imaging

Because the paraffin embedding and microtome sectioning process is largely dependent on the skill of the histotechnologist, optical sectioning was utilized to assess the placement of spheroids within the agar receiver block. In this way, the receiving block is not physically cut, but rather an image is generated by focusing a very thin plane of light within the sample. Images acquired as this plane is moved through the sample can be “stacked” to form a 3-dimensional image. This technique also has the advantage of allowing for rapid “sectioning” of the entire block so that every spheroid can be imaged regardless of planarity.

Optical sectioning was done using a confocal laser scanning microscope. The use of agar for the receiving block had the added convenience of being optically transparent, which is essential for imaging thick samples. Agar receiver blocks were imaged by using a 10x/0.45 plan-apochromat objective to scan an approximately 2.5 cm × 1.5 cm × 1 mm region of the block in a series of 30 μm thick optical sections. To provide contrast between the spheroids and the agar, the spheroids were stained with the HCS CellMask stain which was excited with a 633 nm laser and detected over 634-735 nm. This stain was chosen because it is taken up by the entire cell cytoplasm as well as the nucleus to highlight the entire volume of the spheroids, it can be used with standard Cy5 filter sets, and its longer absorption/emission wavelength penetrates tissue more deeply than the shorter wavelengths of other available stains.

Spheroids were stained after being deposited and embedded in the agar receiver block to increase staining throughput, reduce loss of spheroids during the staining process, and minimize variations in staining between spheroids from the same plate. The HCS CellMask staining solution was prepared as per the manufacturer’s instructions. Prior to staining, the spheroids in each agar receiver block were permeabilized in a solution of 0.1% v/v Triton X-100 in PBS for 1 h. Next, the agar receiver blocks were washed twice in PBS for 30 min each and then soaked in the HCS CellMask staining solution for 1 h. Finally, the agar receiver blocks were washed twice in PBS for 1 h each and stored in PBS until imaging. All solutions and stains were applied in custom Coplin staining jar style containers which were designed to hold the receiver blocks upright so as to allow for adequate diffusion through the top and bottom surfaces of the agar.

### Data analysis and statistics

Image stacks acquired using CLSM were processed using Fiji [19], an open source distribution of the popular image analysis program ImageJ. Using this software, the CLSM data was visualized in 3D, sectioned on arbitrary planes, and analyzed for spheroid position and size. The spheroid distribution planarity was assessed by first computing points on the external surface of all spheroids in a sample. Then for the *i*^*th*^ spheroid, the average distance from the surface of the spheroid to its centroid, ${\overline{r}}_i$, was calculated. Next, a least squares plane was fit to the centroids and the distance from the *i*^*th*^ centroid to the plane, *d*_*i*_, was calculated. This value was then normalized by ${\overline{r}}_i$ yielding the normalized distance to the fit plane $, {d}_i/{\overline{r}}_i$. If the fit plane passes directly through a spheroid’s centroid, then ${d}_i/{\overline{r}}_i=0$. If this value is greater than 1, then it is likely that the fit plane misses the spheroid altogether.

Statistical significance for spheroid success rates averaged over plate rows and columns was established using a one-sample t-test against the total mean success rate (*α* = 0.01 unless otherwise stated). Mean values presented with standard deviation (SD) are shown as mean ± SD.
